# The Reduced Cochlear Output and the Failure to Adapt the Central Auditory Response Causes Tinnitus in Noise Exposed Rats

**DOI:** 10.1371/journal.pone.0057247

**Published:** 2013-03-13

**Authors:** Lukas Rüttiger, Wibke Singer, Rama Panford-Walsh, Masahiro Matsumoto, Sze Chim Lee, Annalisa Zuccotti, Ulrike Zimmermann, Mirko Jaumann, Karin Rohbock, Hao Xiong, Marlies Knipper

**Affiliations:** Department of Otolaryngology, Hearing Research Centre Tübingen (THRC), Molecular Physiology of Hearing, University of Tübingen, Tübingen, Germany; University of Regensburg, Germany

## Abstract

Tinnitus is proposed to be caused by decreased central input from the cochlea, followed by increased spontaneous and evoked subcortical activity that is interpreted as compensation for increased responsiveness of central auditory circuits. We compared equally noise exposed rats separated into groups with and without tinnitus for differences in brain responsiveness relative to the degree of deafferentation in the periphery. We analyzed (1) the number of CtBP2/RIBEYE-positive particles in ribbon synapses of the inner hair cell (IHC) as a measure for deafferentation; (2) the fine structure of the amplitudes of auditory brainstem responses (ABR) reflecting differences in sound responses following decreased auditory nerve activity and (3) the expression of the activity-regulated gene Arc in the auditory cortex (AC) to identify long-lasting central activity following sensory deprivation. Following moderate trauma, 30% of animals exhibited tinnitus, similar to the tinnitus prevalence among hearing impaired humans. Although both tinnitus and no-tinnitus animals exhibited a reduced ABR wave I amplitude (generated by primary auditory nerve fibers), IHCs ribbon loss and high-frequency hearing impairment was more severe in tinnitus animals, associated with significantly reduced amplitudes of the more centrally generated wave IV and V and less intense staining of Arc mRNA and protein in the AC. The observed severe IHCs ribbon loss, the minimal restoration of ABR wave size, and reduced cortical Arc expression suggest that tinnitus is linked to a failure to adapt central circuits to reduced cochlear input.

## Introduction

Tinnitus is a brain disorder causally linked to noise-induced hearing loss, cochlear damage [1], and stress [2], [3], [4], [5], [6], [7]. Due to demographic changes and to increasing use of personal headsets, especially by young people [8], tinnitus is a cumulative challenge. In both tinnitus patients and tinnitus animal models, cochlear damage has been suggested to be associated with subcortical and cortical hyperactivity [1], [9], [10], [11], [12], [13], [14]. Subcortical hyperactivity was observed as increases in spontaneous and evoked spike activity at the level of the dorsal cochlear nucleus (DCN), ventral cochlear nucleus (VCN) and the inferior colliculus (IC) following cochlear damage [12], [15], [16], [17], [18], [19], [20].

To study central responsiveness to auditory trauma related to tinnitus, we used a tinnitus animal model that was designed to minimize stress based on access to sugar water as a positive reward [21], [22]. Unlike most previous studies on tinnitus, we analyzed equally hearing-impaired animals which, based on their behavior, were separated into groups with and without tinnitus [23]. These groups were compared for (i) the number of CtBP2/RIBEYE-positive particles in ribbon synapses of the inner hair cell (IHC) as a measure for deafferentation [24], (ii) the fine structure of the amplitudes of auditory brainstem response (ABR) waves that may reflect crucial differences in sound responses following decreased auditory nerve (AN) activity [25], and (iii) the expression pattern of the rapid immediate early gene Arc/Arg3.1 (activity-regulated cytoskeleton-associated protein/activity-regulated gene 3.1, for simplicity henceforth referred to as Arc) in the auditory cortex (AC).

Arc expression is involved in acute and long-lasting alterations of network activity as a consequence of altered sensory input [26], [27]. Most importantly Arc mobilization is essential for homeostatic adaptation of responsiveness following visual deprivation during development [28], [29]. In order to identify a tinnitus-specific trait rats were exposed to noise and were behaviorally separated into tinnitus and no-tinnitus animals. Animals were analyzed 1–4 weeks after exposure for hearing loss, damage of the IHC synapse, changes in ABR wave amplitude and cortical Arc expression. IHCs ribbon loss (deafferentation) did not lead to tinnitus when brainstem responses were restored and Arc was mobilized in the AC. When brainstem responses remain reduced and Arc was not mobilized, IHC ribbon loss resulted in tinnitus. Both response patterns were found independent of low frequency threshold loss. The results are discussed in the context of a facilitating adaptive (no tinnitus) or non-adaptive (tinnitus) brain response following injury.

## Materials and Methods

In order to identify a tinnitus-specific difference between equally acoustically exposed animals with and without tinnitus (tinnitus-trait), we exposed rats binaurally to noise and behaviorally identified animals with tinnitus. In this model, rats are trained to associate white noise with a sugar water reward and silence with no reward [22]. When rats perceive tinnitus after noise trauma, they incorrectly access the liquid reward because they hear their tinnitus. This access behavior is expressed as silence activity.

To increase the likelihood of detecting a tinnitus-trait, we used various noise conditions. To assure specificity, tinnitus was evaluated in both groups by the same criteria (silence activity >0.1), independently of the hearing loss of individuals. One group of animals was exposed to noise at a sound pressure level of 120 decibels (dB sound pressure level, SPL) at 10 kilo Hertz (kHz) for 1 hour (h) and analyzed 6 days (d) post exposure, when no further recovery of hearing was expected to occur. Another group of animals was exposed to 120 dB SPL for 1.5 h. Since the exposure for 1.5 h resulted in a stronger trauma, we analyzed these animals 30 d post exposure to make certain that the hearing and the tinnitus had recovered at its best and no further recovery of threshold was expected.

Hearing function was studied by ABRs evoked with short acoustic stimuli before and after noise exposure, at the same day as behavioral testing. ABRs represent the summed activity of neurons in the ascending auditory pathway and are measured by averaging the evoked electrical response recorded via subcutaneous electrodes.

### 1.1 Ethics Statement, Animals, and Noise Exposure

Animal care, procedures, and treatments were performed in accordance with institutional and national guidelines following approval by the University of Tübingen, Veterinary Care Unit, and the Animal Care and Ethics Committee of the regional board of the Federal State Government of Baden-Württemberg, Germany (approval number HN5/05).

Adult female Wistar rats weighing 200–300 g were exposed in anesthesia to sound (120 dB SPL), or sham exposed with loud speaker switched off, for either 1 h or 1.5 h, at 10 kHz and sacrificed 6 d, or 30 d after [23].

Animals were anesthetized with a mixture of ketamine–hydrochloride (75 mg/kg body weight, Ketavet 100, Pharmacia, Erlangen, Germany) and xylazine hydrochloride (5 mg/kg body weight, Rompun 290, Bayer, Leverkusen, Germany), injected i.p. Anesthetized rats were binaurally exposed to free field inside a reverberating chamber (a chamber of ca. 50×50×50 cm with tilted, non-parallel walls to avoid standing waves and to achieve a mostly homogeneous sound field). To eliminate furthermore the effects of any possible inhomogeneity in the acoustic field and to avoid that ear were positioned at sites of pressure wave extinction animals were slowly constantly moved through the sound field by means of a rotating turntable and were repositioned on this table in intervals of 30 minutes to ensure a homogeneous exposure of all animals on both ears. The reverberating chamber was equipped with top and side wall mounted speakers (DR48, Visaton, Haan, Germany, and Piezo Horn 335835, Conrad Electronic, Hirschau, Germany). The loudness and spectrum of the traumatizing sound was constantly monitored my means of a microphone placed in the centre of the chamber and customized computer software analyzing the frequency spectrum by fast fourier transformation (FFT). The traumatizing sound had a narrow peak at 10 kHz and sidebands at 20 kHz (−40 dB) and 30 kHz (−60 dB). Additional side bands were below 50 dB SPL and therefore did not exceed the normal laboratory background noise.

Additional doses of anaesthetics were administered if needed and body temperature maintained by heating pads and lamps.

### 1.2 Hearing Measurements

ABRs were measured in a soundproof chamber (IAC 400-A, Industrial Acoustics Company GmbH, Niederkrüchten, Germany) as described [30], [31], [32]. In short, ABRs were recorded in anesthetized adult animals. Electrical brainstem responses to free field click (100 µs, 0–90 dB SPL), and pure tone (1–45 kHz in half-octave steps, 20–100 dB SPL in steps of 5 dB, 3 ms, 1 ms cosine squared rise-fall envelope) acoustic stimuli of alternating polarity (compression and rarefaction) were recorded with subdermal silver wire electrodes at the ear (positive, active), the vertex (negative, reference) and the back of the animals (ground). Recordings were made for 10 ms with stimulus presentations of alternating polarity to eliminate electrical artefacts. In each case, stimulus presentation was at time 0 ms. The click stimulus was a broadband stimulus with a center frequency at 4.9 kHz (50th percentile) and the 25th and 75th percentiles at 2.2 kHz and 13.8 kHz, respectively. Signals were amplified (50,100-fold, 94 dB), bandpass filtered (0.2–5 kHz 6-pole Butterworth filter, Wulf Elektronik, Frankfurt, Germany), averaged across 64–256 repetitions (dependent on the signal to noise ratio, but always the same repetition time at close threshold stimulation) at each sound pressure presented (usually 0–100 dB SPL in steps of 5 dB), and recorded at 10 kHz sample frequency. Stimuli were delivered to the ear in a calibrated open system by a loudspeaker (DT-911, Beyerdynamic, Heilbronn, Germany) placed 3 cm lateral to the animal’s pinna. Sound pressure was calibrated online prior to each measurement with a microphone (B&K 4191, Bruel & Kjaer, Naerum, Denmark) placed near the animal’s ear.

For stimulus generation and recording of ABRs, a multi-function IO-Card (PCI-6052E or PCI-MIO-16E1, National Instruments, Austin, Texas, USA) was used, housed in an IBM compatible computer. Sound pressure level was controlled with an attenuator and amplifier (Wulf Elektronik). Filter settings, ABR spectral components (by FFT), potential stimulus artefacts were examined beforehand to ensure that ABR signal waveform, amplitude and latencies of distinct peaks in the ABR were not affected by the chosen hardware and software parameters. In particular, to reduce physical stress of the animals by long lasting anaesthesia to a minimum, ABR measurement times were reduced to a minimum by increasing stimulus repetition rates to 80/s, minimizing repetition numbers for clearly suprathreshold signals (when ABR wave amplitudes were exceeding ±4 µV), and reducing sample rates to 10 kHz to reduced delay times by computer online analyses. In control studies it was validated that the protocols that were finally used (64–256 repetitions, 0.2–5 kHz bandpass filtering, 10 kHz sampling rate), gave homogeneous results as compared to higher repetition numbers (512) and higher (100 kHz) sample rates.

Hearing threshold was determined by the lowest sound pressure that produced visually distinct evoked potentials from above threshold to near threshold.

### 1.3 ABR Analysis

For each individual ear, the auditory brainstem response (ABR) waveform to click stimuli (*waveform analysis*), and the peak input-output function (*peak I/O*) was analyzed.

#### Waveform analysis

The ABR wave functions of individuals were averaged. Average curves were built for four different groups: (1) no-tinnitus rats before exposure, (2) no-tinnitus rats after exposure, (3) tinnitus rats before exposure, and (4) tinnitus rats after exposure. For all four groups, the ABR functions were analyzed at a stimulus level of 90 dB SPL (a high stimulus level) and at 40 dB above hearing threshold (40 dB hearing level). The average waveforms gave a qualitative and temporal assessment of changes in amplitude ranges in the ABR function.

#### Peak I/O analysis

The ABR wave data for the click stimuli were analyzed for peak and through amplitudes and the latencies by customized computer programs.

From individual ABR waves to click stimuli, peak amplitudes and peak latencies were collected, grouped in clusters of similar peak amplitude and latencies, and averaged for ABR wave input-output (I/O) analysis. Wave amplitudes were defined as peak to peak amplitude of a negative peak (n) followed by a positive (p) peak. Clusters of peaks were found at average latencies n0.9-p1.2, n1.3-p1.6, n1.9-p2.4 or p2.7, n3.6-p4.9, and n7.1-p9.4. The ABR recordings are presented for the first 10 ms recording time. To affirm that the peak at p9.4 was not missed out due to a peak latency longer than 10 ms, the whole recording cycle consisting of a compression and a rarefaction stimulus (lasting 20 ms, see above: hearing measurements) was analyzed and the peak position approved within the first 10 ms. For selected peaks and troughs the I/O functions were derived from the peak-to-peak amplitudes at all recorded stimulus sound pressure levels. Three peak classes were selected: (1) early peaks (at 0.9–1.2 ms, with the “wave I” interpreted as the sum of the first stimulus-related action potential within the auditory nerve); (2) delayed peaks (at 3.6–4.9 ms), found in the range of the greatest loss of the ABR waveform (as determined by analyzing the square difference between ABR curves before and after noise exposure, not shown); and (3) late peaks (at 7.1–9.4 ms), as these peaks fall into the time range of thalamic activation.

#### Waveform correlation analysis

The similarity in the waveform of the ABR before and after an acoustic noise exposure is founded on changes of all peak amplitudes and all peak latencies. In human tinnitus studies, often the relation of wave-I to wave-V is reported [33]. Since these parameters are circumstantial to describe in the rat ABR data, we applied a general measure for the “similarity” of ABR waves: As an estimation for the recovery of click-ABR waves after acoustic noise exposure, correlation of ABR waves before and after recovery (6–30 d) were analyzed by the correlation factor (CorF):
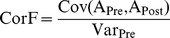
where Cov: covariance of the click-ABR waves before exposure and after recoveryA_Pre_: the amplitudes at point 1 to 100 (0–10 ms) before exposureA_Post_: the amplitudes at point 1 to 100 (0–10 ms) after exposureVar_Pre_ variance of the click-ABR wave before exposureCorF: correlation factor (covariance/variance) 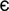 R

The CorF therefore provides a normalization of the covariance with respect to the variance of the measurement before exposure. In contrast to the correlation by the Pearson Momentum Coefficient that is not sensitive to overall amplitude changes, the CorF reflects changes in waveform and amplitude. Typical values are 1.0 for optimal correlation between waveforms of similar amplitude values. Loss of similarity and loss of amplitude typically result in CorF values approaching null.

### 1.4 Behavioral Animal Model

Within 3–4 months before the noise exposure, rats were trained using operant conditioning to perform a specific motor task (foraging behavior for sugar water, on average 1–3 timely randomized rewards per minute) when perceiving an external sound cue (presented for 1–3 minutes) and to cease this motor activity during periods of silence (lasting 40–120 seconds, accesses during silence were randomly paired with electrical foot shocks of 0.1–0.4 mA, 100 µs). A conditioning session lasted 20–60 minutes containing 10–30 silence and sound periods, sound stimuli were varied between 70 and 10 dB SPL (in steps of 10 dB), and 32–38 sessions were required to train the rats to meet the training criterion. Correct indication of tinnitus of either rats was tested by transient tinnitus induction after salicylate injection (350 mg/kg bodyweight) [22] In test situation, rewards and foot shocks were omitted and sound (broad band noise sound of 60 dB SPL) and silence periods (60 s each) were presented interleaved for 23 minutes, as described [22]. Animals with tinnitus actively execute the motor task even when the external sound cue has not been presented, for details see [22]. A detailed protocol for noise-induced tinnitus is described in [23]. The motor task was quantified by the ratio of activity during external sound and the activity during periods of silence (silence activity). Typical values for silence activity are below 0.1 for no-tinnitus and above 0.1 for tinnitus animals, dependent on the conditioning level. White noise sound stimuli were presented for operant conditioning and testing in the experimental phase. These stimuli could easily be generalized by rats with their induced tinnitus percept, even if (for our particular method of induction) this tinnitus percept was of unknown frequency and loudness.

### 1.5 Tissue Preparation

Cochlear and brain tissues were dissected as previously described [23]. For detection of mRNA and protein, brains were fixed for 48 h in 4% paraformaldehyde, embedded in 4% agarose and stored at 4°C. The tissue was sectioned at 60 µm with the Leica VT 1000S vibratome (Wetzlar, Germany).

### 1.6 Immunohistochemistry and Ribbon Counts

Rat cochleae were isolated, fixed, cryosectioned, and stained as described in [23]. Mouse monoclonal anti-CtPB2/RIBEYE antibody (BD Transduction Laboratories, San Jose, CA, USA) and rabbit polyclonal anti-GluR4 antibody (Millipore, Schwalbach, Germany) were used as primary antibodies. Image acquisition and CtBP2/RIBEYE-immunopositive spot counting were carried out as previously described [34]. In brief, cryosectioned cochleae were imaged over a distance of 8 µm covering the entire IHC nucleus and areas beyond it in an image stack along the z-axis (z-stack). One z-stack consisted of 30 layers with a z-increment of 0.28 µm; for each layer, one image per fluorochrome was acquired. To display spatial protein distribution, z-stacks were three-dimensionally deconvoluted using Cell F’s RIDE module with the Nearest Neighbor Algorithm, Voxel Viewer, and Slice Viewer (Cell F, OSIS 231 GmbH, Münster, Germany).

### 1.7 Co-localization of mRNA and Protein in Brain Sections

Riboprobes were designed as described in [23]. mRNA and protein were co-localized on free-floating sections. Following pre-hybridization for 1 h at 37°C, sections were incubated overnight with Arc riboprobes at 56°C, incubated with anti-digoxigenin antibody conjugated to alkaline phosphatase (anti-Dig-AP, Roche, Mannheim, Germany) and developed as previously described [35]. For protein detection, streptavidin-biotin was blocked according to the manufacturer’s instructions (Streptavidin-Biotin Blocking Kit, Vector Laboratories, Burlingame, CA, USA) after blocking endogenous peroxidase. Sections were incubated overnight at 4°C with the primary antibodies against Arc (Synaptic Systems, Göttingen, Germany), followed by incubation with the secondary antibody (biotinylated goat anti-rabbit, Vector Laboratories) and chromogenic detection (AEC, 3-amino-9-ethylcarbazole, Vector Laboratories). Sections were counterstained with the nuclear marker methyl-green (Vector Laboratories), embedded with gelatin, and analyzed using an Olympus AX70 microscope.

### 1.8 Data Analysis

#### Statistical analysis

Correlation factors and hearing loss were compared using Student’s t-Test. The statistical significance at the alpha level of 0.05 is indicated in the figure legends.

Tinnitus behavior was compared using the Mann-Whitney-U Test. For the ribbon counts of apical, medial and basal cochlear turns, P-values were corrected for alpha-shift by multiple testing using the Bonferroni-Holms procedure.

Ribbon numbers from 3–5 animals from 3 independent experiments per group were counted and compared by 1-way ANOVA. For the ribbon counts of apical, medial and basal cochlear turns, P-values were corrected for alpha-shift by multiple testing using the Bonferroni-Holms procedure.

#### Quantification of Arc/Arg3.1 positive cells in the AC

Arc mRNA expressing cells were counted in the AC as previously described [35]. In brief, Arc immunoreactive cells were counted in the AC of coronal brain sections using an integrated microscopic counting chamber to fix the area of interest delineated by a square of 2,450 µm2. The number of Arc positive cells from eight 2,450 µm2 squares on four rat brain sections for each treatment group (between 4.2 and 5.2 mm posterior to Bregma) [36] including the 10 kHz region of the auditory cortex were counted throughout the thickness of the slice, and the average was taken. These squares were placed with respect to the cellular anatomy of the AC: they covered cortical layers II to VI and spanned the bulk of the area designated as the primary AC according to Doron et al. [36]. Data are expressed as mean cell count ± S.E.M. Statistical analysis was performed using the two-tailed Student’s *t* test, with alpha = 0.05. Cells were counted in 3 animals per group in three independent experiments.

## Results

**Figure 1:**
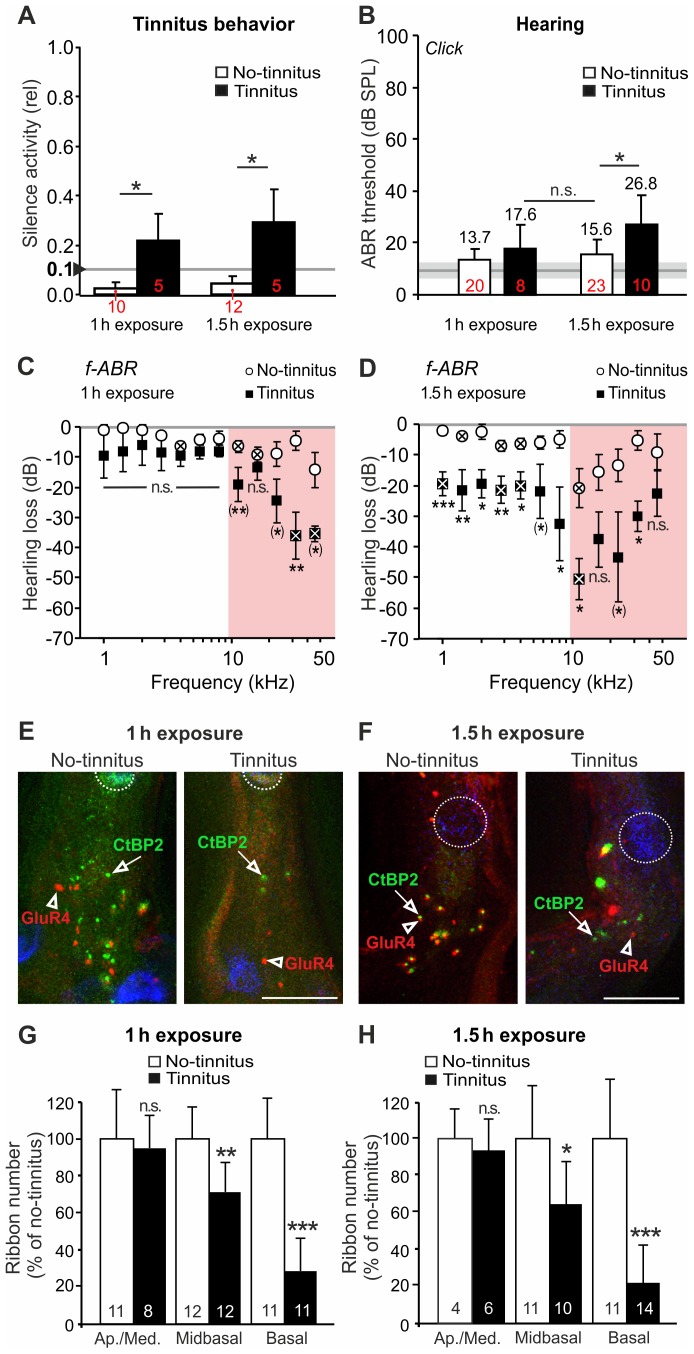
Development of tinnitus in equally sound-exposed animals correlates with altered structure of the IHC synapse. (**A**) Following exposure to 120 dB SPL at 10 kHz for 1 h (assessed at 6 d after exposure) or for 1.5 h (assessed at 30 d after exposure), tinnitus occurred in ∼30% of the animals. Mean silence activity (± S.D.) as a measure for tinnitus for no-tinnitus (white) and tinnitus animals (black). The criterion for tinnitus was a silence activity above 0.1 (horizontal grey line, black triangle on ordinate). Number of animals is given in or below the bars. (**B**) A significant difference in the hearing threshold to click stimuli was only observed between animals with or without tinnitus after 1.5 h exposure. Mean (± S.D.) ABR thresholds for click stimuli of no-tinnitus (white) and tinnitus animals (black), tinnitus judged from their silence activity. Hearing threshold is depicted above each bar. Number of animals is given within each bar. The grey horizontal line and area illustrates the mean ABR threshold (± S.D.) before exposure. (**C, D**) High-frequency hearing is impaired following noise exposure, more pronounced in tinnitus animals than in no-tinnitus animals. Following 1.5 h exposure (**D**), also low-frequency hearing is impaired in tinnitus animals. Mean ABR threshold loss (dB, ± S.E.M.) to frequency-specific tone bursts for no-tinnitus (circles) and tinnitus animals (squares). The grey line at the top of each panel shows the normal hearing threshold before exposure. Frequencies with a significant hearing loss (>99% confidence interval of hearing threshold before exposure) are marked by crossed symbols. Frequencies with a significantly different loss between no-tinnitus and tinnitus animals are indicated by asterisks (Student’s t-Test with Bonferroni-Holms adjustments for alpha-shifts). Asterisks in brackets indicate descriptive statistics for p-values from t-tests that fail to meet the Bonferroni-Holms criterion. n.s. not significant. (**E, F**) Antibody staining for GluR4 (red, open arrowhead) and ribbon synapses (CtBP2, green, open arrow) are shown for the IHCs of the midbasal turn for the animals used in (A, B). Cell nuclei are stained with DAPI (blue). Scale bars, 10 µm. (**G**) IHC ribbon numbers of control and no-tinnitus animals were not significantly different. IHC ribbon numbers of hearing-impaired tinnitus animals were significantly reduced in the midbasal and basal turns in comparison to no-tinnitus animals. Ribbon counts were compared for statistical significance using the 1-way ANOVA, p-values were corrected for alpha-shift by multiple testing using the Bonferroni-Holms procedure, df = 8.

Behavioral testing [22], [23] showed that ca. 30% of the animals in both groups (5 of 15 rats and 5 of 17 rats for the 1 h and 1.5 h exposure, respectively) had developed a significantly elevated silence activity, indicating occurrence of tinnitus (Fig. 1A, difference for both groups: p<0.02 for the Mann-Whitney-U Test).

ABR thresholds for click-stimuli (Fig. 1B, Table 1A) and frequency-specific stimuli (Fig. 1C and D, Table 1A) revealed a permanent, though mild, threshold loss in all animals that increased with exposure duration (Table 1A). ABR threshold loss for click stimuli was mild but significant for all groups (p = 0.0115, 0.0403, 0.0074 and 0.0004 for 1 h no-tinnitus, 1 h tinnitus, 1.5 h no-tinnitus, and 1.5 h tinnitus, respectively for the Student’s t-Test). This indicates that a hearing loss to broadband click stimuli was not necessarily leading to tinnitus (studied by behavioral testing). Since the click stimulus contained frequencies predominantly in the lower frequency range of the hearing range of a rat (1–10 kHz), a loss of these frequencies may not be of relevance for inducing tinnitus. However, the group of tinnitus rats exposed to 1.5 h had a significantly larger hearing loss than no-tinnitus rats (Table 1A, 18.20±4.23; n = 5 for tinnitus rats), and also individual animals in both no-tinnitus groups showed significant hearing loss at isolated low frequencies (Fig. 1 C, D, crossed circles), what raises the question how hearing loss for low and high frequencies might contribute to the generation of tinnitus. To specify the hearing function in the high frequency hearing range of the rats we performed frequency specific ABR measurements. When frequency-specific ABR thresholds between tinnitus and no-tinnitus groups were compared, we consistently observed that in both tinnitus groups, hearing loss for frequencies above 11.3 kHz was significantly increased compared to no-tinnitus groups (Fig. 1C and D; Table 1A). After the more intense noise exposure (1.5 h), threshold loss in tinnitus animals was also significantly greater for low stimulus frequencies (Fig. 1D, *: p<0.05, **: p<0.01, ***: p<0.001, Student’s t-Test with alpha correction for multiple comparison).

**Table 1:** Hearing loss in no-tinnitus and tinnitus animals.

	Click-ABR		F-ABR >11.3 kHz	
	1 h, 120 dB SPL	1.5 h, 120 dB SPL	1 h, 120 dB SPL	1.5 h, 120 dB SPL
**No-tinnitus**	3.4±3.2; n = 10	6.1±4.2; n = 12	7.9±5.6; n = 10	11.5±10.6; n = 12
**Tinnitus**	7.4±5.5; n = 5	18.2±8.1; n = 5	22.8±8.5; n = 5	38.7±15.1; n = 5
**Significance**	n.s.	***	**	**

Hearing loss (in dB) in no-tinnitus and tinnitus animals following 1 h or 1.5 h exposure using click- and frequency-specific stimuli. Animals were either exposed to 120 dB SPL, 10 kHz for 1 h and analyzed after 6 d or exposed for 1.5 h and analyzed after 30 d. The groups are subdivided in tinnitus animals and no-tinnitus animals. A significant difference in hearing threshold was observed between no-tinnitus and tinnitus animals following exposure at stimulus frequencies of 11.3 kHz and above (*: p<0.05, **: p<0.01, ***: p<0.001).

**Table 2:** Number of inner hair cell ribbons in no-tinnitus and tinnitus animals.

	unexposed	1–1.5 h, 120 dB SPL		
	Control	No-tinnitus	Tinnitus	P
**Apical/Med.**	16.4±3.5	15.4±2.4 (n.s.)	14.6±1.2 (n.s.)	n.s.
**Midbasal**	20.7±4.0	18.6±2.8 (n.s.)	12.6±2.7 (***)	*
**Basal**	18.8±3.5	15.5±3.1 (n.s.)	4.3±2.4 (***)	***

Average number of ribbons counted in IHCs of indicated cochlear turns from 3–5 animals (corresponding to animals measured in A) from 3 independent experiments. Statistics in brackets indicate differences in comparison to control, P indicates differences between no-tinnitus and tinnitus animals (n.s.: not significant, *: p<0.05, **: p<0.01, ***: p<0.001).

Although the various intensities of sound exposure led to a variable amount of hearing loss, the 1 h exposed tinnitus rats and the 1.5 h exposed tinnitus-free rats had a similar low frequency hearing function (Fig. 1B), though both groups had significant frequency specific hearing loss after noise exposure. This suggests that threshold loss at low frequencies per se is not leading to tinnitus. Animals with tinnitus had a characteristic threshold loss in the high-frequency regions.

### 2.1 Tinnitus and No-tinnitus Animals Differ in their Degree of Damage at the IHC Synapse

We counted IHC ribbons from 3–5 animals per group (unexposed, no-tinnitus, tinnitus) as a correlate of the number of afferent auditory fibers [24], [37] using antibodies directed against CtBP2/RIBEYE in combination with a postsynaptic marker, the glutamate receptor isoform 4 (GluR4) [24] (Fig. 1E and F). Similar to previous studies in mice [24], the highest number of ribbons in rats was detected in the midbasal cochlear turn (Table 1B). Number of IHC ribbons for unexposed control and no-tinnitus animals were not significantly different (Fig. 1 G, Table 1 B). Animals with tinnitus showed a significantly stronger loss of ribbons (up to 82%), most pronounced in the basal turn, covering frequencies above 17 kHz, and midbasal turn, covering frequencies above ∼11 kHz [38] (Fig. 1G show ribbon numbers in percent compared to control rats, Table 1B shows absolute values, n = 35, done in triplicate, *: p<0.05, **: p<0.01, ***: p<0.001, 1-way ANOVA). In the apical and medial cochlear turns, ribbon loss was not significantly different between animals with or without tinnitus (Fig. 1G, Table 1B, n.s., not significant, Mann-Whitney Test). The loss of afferent fibers was confirmed qualitatively by the reduced expression of postsynaptic GluR4 in animals with tinnitus as compared to no-tinnitus animals (Fig. 1E and F).

These data indicate a severe damage of the IHC synapse in high-frequency cochlear turns of animals with tinnitus in comparison to tinnitus-free animals with similar hearing thresholds for click auditory stimuli (Fig. 1B).

### 2.2 Tinnitus and No-tinnitus Animals Differ in their ABR Wave Size (Synaptic Responses) Following Auditory Deprivation

**Figure 2:**
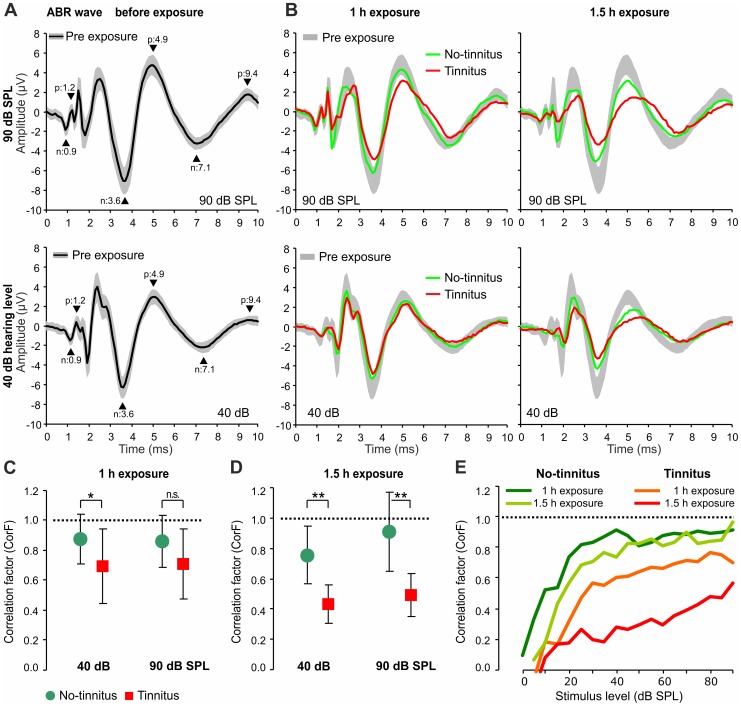
Changes in ABR waveforms following noise exposure is more pronounced in animals with tinnitus. (**A**) Average ABR waveform before exposure at stimulation level 90 dB SPL (upper panel) and 40 dB above hearing threshold (lower panel). Mean (black line) ± S.D (grey area) of n = 32 animals. (**B**) ABR waves illustrating the difference in ABR waveform after 1 h (left panels) or 1.5 h (right panels) noise exposure for animals with (red) or without tinnitus (green) in comparison with the waveforms before noise exposure (mean ± S.D., black line and grey area) depicted for 90 dB SPL (upper panels) and 40 dB above the hearing threshold (lower panels). **(C)** Correlation of ABRs to click stimuli before and after 1 h noise exposure of individual animals (expressed as the correlation factor (CorF) for close to threshold (40 dB hearing level) and at high stimulation levels (90 dB SPL). Mean (± S.D.) derived from n = 10 (No-tinnitus, green circles) and n = 5 (Tinnitus, red squares) rats. The correlation factor of 1 (dashed horizontal line) indicates a perfect similarity of ABR waveforms before and after noise exposure. Correlation was significantly lower in tinnitus animals at 40 dB. At 90 dB SPL, the difference was not quite significant (p = 0.055). (**D**) Correlation of ABRs to click stimuli before and after 1.5 h noise exposure. At both 40 dB and 90 dB SPL, the correlation factor of ABRs from animals with tinnitus was significantly reduced. n = 12 and 5 for no-tinnitus and tinnitus animals, respectively. * p 0.05, ** p<0.01, 1-sided t-Test. (**E**) Correlation of averaged ABRs to click stimuli as a function of stimulus levels (dB SPL). In all four groups, the correlation factor steeply increased at supra-threshold levels. For tinnitus animals, the ABR waveform correlation did not reach the value of no-tinnitus animals, indicating reduced amplitude and waveform recovery after noise exposure.

IHC ribbons determine the generation of spikes in afferent auditory fibers [39]. The summed activity of the auditory nerve is determined by the synchronicity and the reliability of spikes within active fibers [40]. This activity propagates in the ascending auditory pathway and generates the ABR waves in the ventral cochlear nucleus (VCN, wave II, Fig. 2, <p1.2), superior olivary complex (SOC, wave III, Fig. 2<p3.6), responses in the lateral lemniscus and the IC (wave IV, Fig. 2>p4.9) as well as the IC output activity (wave V, Fig. 2>p7.1) [41].

Click-evoked ABR waveform amplitudes before and after noise exposure were compared for differences in signal amplitudes for 1 h (no-tinnitus: n = 10 animals; tinnitus: n = 5 animals) and 1.5 h exposure duration (no-tinnitus: n = 12 animals; tinnitus: n = 5 animals) at 40 dB above threshold and 90 dB SPL (Fig. 2A and B). These conditions allow to distinguish between response amplitudes derived from low-threshold fibers (∼60%) with a high spontaneous rate (high-SR) that have a fast saturation at about 40 dB SPL, and amplitudes from high-threshold fibers (∼40%) with a low spontaneous rate (low-SR) that respond to higher SPLs [42], in this case to a 90 dB SPL stimulus.

As expected from the permanent, although mild, hearing loss to click stimuli within both groups (Fig. 1B, Table 1A), a reduction in the overall amplitude of the sound-evoked signals compared to the waveform prior to exposure was observed in animals with and without tinnitus (Fig. 2A and B) for 90 dB SPL (upper row) and 40 dB (lower row). For both stimuli conditions the correlation analysis of the ABR waves (CorF) revealed a reduced recovery for tinnitus animals in comparison to tinnitus-free animals (Fig. 2C and D) across the whole input/output (I/O) range of stimulus levels (Fig. 2E).

**Figure 3:**
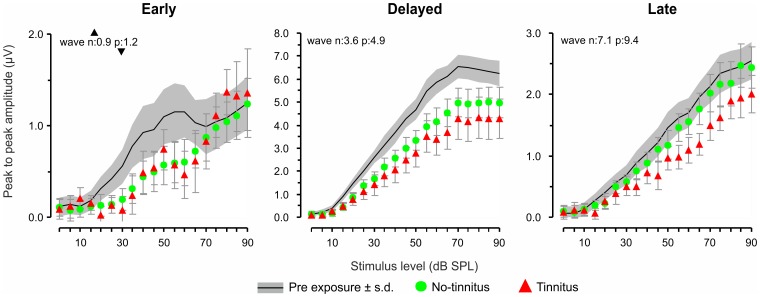
Peak-to-peak amplitudes of late peaks of ABR waves remain reduced following noise exposure in animals with tinnitus. Mean peak growth input/output (I/O) function (± S.D.) for early, delayed and late peaks before exposure (black line and grey shaded area) after 1 h or 1.5 h exposure. Three selected peak-to-peak amplitude growth functions (µV) with increasing stimulus levels (dB SPL) are shown for rats with tinnitus (green) or without tinnitus (red). In the rats with tinnitus, the peak-to-peak amplitudes remain reduced up to late peaks (right panel). The peak latencies are given in each panel for negative (n) and positive (p) peaks.

This suggests that both, high-SR, low level and low-SR, high level auditory fibers might be affected. This was studied by the I/O growth of the dominant peaks (indicated in Fig. 2A, arrowheads) before and after exposure (Fig. 3). Importantly, the amplitude waves of the early peaks (Fig. 3, Early), corresponding to the AN or cochlear nucleus (CN), were reduced in animals with and without tinnitus following both exposure protocols (1 h or 1.5 h, 120 dB SPL, 10 kHz). ABR amplitude wave size remained reduced particularly at lower threshold levels up to 60 dB SPL (Fig. 3, Early), indicating that responsiveness of afferent fibers with both high-SR (low-threshold) and low-SR (high-threshold) are affected.

We need to point out that click stimuli are used that are dominated by frequencies lower than 10 kHz (see methods 2.2). As shown by [43] for cats following middle to high frequency noise trauma, units with low characteristic frequencies (CFs) still respond at threshold, though amplitudes of compound action potential (CAP) responses are reduced. In accordance, we found a moderate ABR threshold loss using the click stimuli which are predominately stimulating cochlear regions below 10 kHz. We therefore would not expect to find differences of early peak amplitudes (ABR wave I) between tinnitus and no-tinnitus rats.

However, for tinnitus-free animals, amplitude functions improved at delayed peaks and showed nearly complete recovery at late peaks compared to animals with tinnitus (compare Fig. 3, *Delayed* and *Late*). This suggested that in tinnitus-free animals, responsiveness to sound at the level of the lateral lemniscus and inferior colliculus (IC) (*Delayed*) or at the level of the IC output and medial geniculate body (MGB) (*Late*) is enhanced, in comparison to animals with tinnitus. As cochlear CF regions of best hearing of the animals are the main source of ABR waves generated in higher brain regions (wave V, [44], the difference in high-frequency CF regions above 11.3 kHz between tinnitus-free and tinnitus animals is likely the drive for less or more elevated late peaks in higher brain regions of these animals. Pure tone stimuli with frequencies above 10 kHz would be expected to result in differential growth function also in early peaks between tinnitus and no-tinnitus animals. In future studies this aspect will be analyzed in detail. However, due to the hearing loss at higher frequencies, ABR wave I response for high-frequency and moderate sound pressure stimulation is expected to be small in case of trauma.

### 2.3 Tinnitus and No-tinnitus Animals Differ in Arc Levels in Sensory-deprived Cortical Regions

To further analyze to what extent the differences in ABR wave sizes may be linked to gain in central auditory circuits, we studied the expression of Arc mRNA and protein to trace neurons that are long-lastingly activated [26], [27], [45]. We used a method that permits the simultaneous monitoring of expression changes in Arc mRNA and protein.

**Figure 4:**
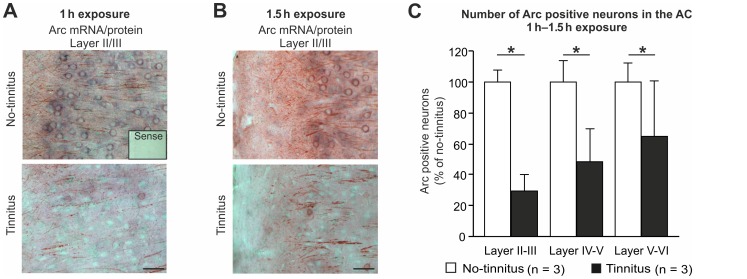
Silencing of Arc expression in auditory cortex (AC) in animals with tinnitus. (**A, B**) Double detection of Arc mRNA (blue) and Arc protein (red) in the AC of equally noise-exposed rats shows a significantly reduced expression in animals with tinnitus in all cortical layers, quantified in **(C**, unpaired Student´s t-test, p<0.001, alpha = 0.05, df = 6**)**. Scale bars, 50 µm. n = 3 animals per group in three independent experiments. Images correspond to coronal sections 2.5 and 3.6 mm posterior to Bregma. Hybridization with sense riboprobes plus omission of the primary antibody produced no signals (insert in A, Sense).

A significant decline of Arc expression in neurons was found in all layers of the AC of animals with tinnitus monitored following both exposure protocols (1 h, 120 dB SPL, 10 kHz, Fig. 4A and C and 1.5 h, 120 dB SPL, 10 kHz, Fig. 4B and C) in comparison to no-tinnitus animals (n = 3 animals, p<0.001 for unpaired Student’s t-test, α = 0.05).

The observation that Arc expression levels are reduced in the AC only in those animals that also exhibit reduced IHC ribbon numbers, reduced ABR wave size and tinnitus, strengthens the argument that animals with tinnitus have developed a rather reduced (instead of enhanced) responsiveness of central circuits.

## Discussion

The present study compared markers for deafferentation, brainstem responsiveness and homeostatic plasticity in equally acoustically exposed animals that were behaviorally separated into groups of animals with and without tinnitus. A characteristic pattern of severe IHCs ribbon loss, insufficiently restored late ABR wave and a failure to mobilize Arc in the cortex could be linked to high-frequency hearing impairment and tinnitus. This finding is discussed in the context of a failure to recruit central gain following cochlear deprivation as a correlate of tinnitus.

### 3.1 Behaviorally-tested Tinnitus is Associated with IHC Ribbon Loss

To assure the specificity of a tinnitus-specific trait, this trait should be a discrete property, independent of hearing loss. The rats with tinnitus after a mild trauma (1 h) and the rats in the no-tinnitus group after mild (1 h) and a more extensive trauma (1.5 h) had a similar low frequency hearing. This indicates that the amount of threshold loss required for tinnitus induction is not necessarily of a uniform dimension, as already suggested by other authors [46], [47], [48], [49]. Indeed various investigations show tinnitus to occur even when hearing impairment cannot be detected by hearing threshold tests [47], [50], [51].

Irrespective of the amount of hearing impairment, we observed that tinnitus was coupled to severe IHC ribbon loss after both exposure durations in 30% of animals, an incidence also observed among hearing-impaired humans [1] but different from the 50–70% of tinnitus animals depicted using gap-detection methods [16], [52].

### 3.2 Differential IHC Ribbon Loss is Linked to Different ABR Wave Size in Higher Brain Regions

Approximately 20 ribbons tether synaptic vesicles in the active zones of an IHC [53], each driving a postsynaptic AN fiber [54] and determining through the maintenance of a large releasable pool of synaptic vesicles the reliability and precision of spikes [39]. Typically, the spike response of AN runs through a maximal rate before approaching adapted rates [55]. As peak rates influence ABR thresholds, severe ribbon loss would lead to worsened thresholds despite intact outer hair cell (OHC) function [39]. This would explain why tinnitus animals with severe ribbon loss exhibit higher ABR thresholds in affected regions (Fig. 1C and D). ABR threshold loss in high-frequency regions was also detected in subjectively normal-hearing tinnitus patients [56].

Using click stimuli spectrally dominated by frequencies below 10 kHz, we found mild and similar but significant hearing loss (3–18 dB, Fig. 1B, Table 1A) and ABR wave I reduction in tinnitus and no-tinnitus animals (Fig. 3), indicating that units with low characteristic frequencies (CFs) were only slightly affected following moderate noise trauma, independent of a strong damage of units with high CFs, as also shown for cats [43]. High CF regions of the cochlea participate in the generation of ABR waves in higher brain regions [44] and may thus be directly linked to differences in ABR wave size at delayed (lateral lemniscus, IC) and late peaks (IC output, MGB) in animals with tinnitus (Fig. 3). The 82% ribbon loss in animals with tinnitus must include low-threshold afferent fibers with high-SR since this fiber class comprises 60% of afferent fibers [42]. A loss of high-SR fibers has been already previously observed in tinnitus studies [46]. Based on computational models of tinnitus development [15], [57], [58], it was hypothesized that deafferentation of a substantial fraction of the AN fibers, as observed in mice following “temporary” hearing loss [24], could trigger the development of elevation of central spontaneous firing rates [57], [58], [59]. This elevation of central spontaneous firing rates following deafferentation would be accompanied by enhancement of neural synchrony [57] in the ascending auditory pathway leading to increased central gain, as suggested in a `gain adaptation model` for tinnitus [15]. This model suggests that increased central gain in the frequency range that is affected by hearing loss [60] is generated through deficit in AN function manifested as a reduction in nerve output at high sound levels, indicating deafferentation of high-threshold low-SR AN fibers [17]. Whether increased central gain fails to be generated when a critical number of low-threshold high-SR AN fibers is lost, as suggested in the present study to occur in tinnitus animals, needs to be analyzed in more detail in future studies.

The restored ABR waves observed here in hearing impaired animals without tinnitus mainly reflect spreading activity through the VCN but not DCN [41]. Higher discharge rates generated in the VCN following acoustic trauma have been shown to lead to steeply increasing loudness recruitment during hyperacusis [61], [62], [63]. We therefore hypothesize that the differences in Delayed and Late ABR waves observed here between tinnitus and tinnitus–free animals reflect differences in compensating increases in VCN activity between tinnitus and hyperacusis animals both occurring as a consequence of reduced cochlear input [61].

As our results show that ABR waves are restored in the tinnitus-free situation, the findings do not support the idea that increased responsiveness in CN target neurons is a correlate for tinnitus. [17], [60]. In this context it must be considered that previous studies never compared the central responsiveness in equally traumatized animals, and so far no patients with hearing loss (reduced wave I) but without tinnitus have been studied [17].

The more restored ABR waves in tinnitus-free animals may reflect a form of loudness recruitment or hyperacusis and the less restored ABR waves in tinnitus animals a reduced capacity to compensate missing gain in the ascending pathway. Data would thereby experimentally support a recent computational model that suggests steeper rate level function in brainstem neurons as the source of non-linear gain that produces loudness recruitment and hyperacusis [64]. Accordingly, in patients with both tinnitus and hyperacusis, steeper than normal loudness growth functions were found, while tinnitus patients without threshold loss had normal loudness growth at the tinnitus frequency [65].

### 3.3 Reduced ABR Wave Size is Correlated with Reduced Arc Levels in the AC

In the cortex, Arc mRNA is expressed in non-GABAergic glutamatergic neurons [26], [27], [66], [67], [68], [69].

Arc mRNA is directly transported within minutes to distal dendrites following LTP like activity [70], where through sustained translation for 2–4 h, Arc protein scales surface AMPA receptors in dendritic spines up and down, a process essential for LTP consolidation, for review see [27], [45], [70], [71], [72], [73], [74]. Moreover, Arc mediated synaptic scaling is an essential need for a system to respond to continuous changes in activity, maintain averaged firing rate [75], [76], [77] and regulate cell-wide responses to long-term changes in activity including responses to reduced input [78] or e.g. visual deprivation [29]. (see for a review [45]. Arc mobilization has also been described in pyramidal neurons of layer II-III following e.g. environmental enrichment [79] Furthermore, Arc mobilization is linked to higher density of dendritic spines [79] or increased sensitivity to glutamate and synaptic strength [28].

Since Arc protein is rapidly degraded [27] the long-lasting changes in Arc mRNA and protein observed in previous studies [80], [81], [82] and in the present study likely reflect permanently altered network activity.

While Arc mobilization in the cortex may elevate the sensitivity of cortical pyramidal neurons [28] the sensitivity after the failure to mobilize Arc, as observed here in tinnitus animals, remains to be explored. Plasticity, specifically homeostatic scaling, is highly sensitive to Arc levels. The scaling responses to manipulation of neuronal activity in vitro are lost in Arc KO neurons [73] and loss of Arc is disrupting the ability of spine formation [83]. Arc deletion has been shown to lead to an increase in basic mEPSCs [29] and development of highly synchronized epileptic-like cortical network activity [84]. High synchronization and epileptic-like neuronal activity in sensory-deprived frequency regions of the primary auditory cortex are also assumed to be associated with cortical activity changes during tinnitus [51], [85], [86], [87]. Arc decline has been observed in cortical regions with reduced thalamo-cortical input in regions >8 kHz (determined by cortical field potentials) [23] tonotopically related to the regions where a decline of IHC ribbon numbers in the cochlea occurred (Fig. 1 G and F, see also [88] and where the most pronounced hearing deficit was observed (Fig. 1 C and D). The failure to mobilize Arc could explain the perception of the tinnitus pitch within the frequency deprived region [51], [89]. Since we found a reduction of IHC ribbons and ABR waves that correlate with reduced Arc levels in the AC as a feature of tinnitus, we may conclude that potentiating activity essential to drive Arc mobilization [70], [73], [74] is obviously missing in the frequency deprived region during tinnitus.

The rapid deafferentation [24], [90], [91], and the rapid changes of Arc mobilization [27], [45] would moreover be in line with the immediate and transient appearance of tinnitus and hyperacusis [92], [93], [94], [95], [96], [97].

### 3.4 Conclusion

In conclusion, the current findings provide a rationale for the altered responsiveness of central circuitries after different degrees of IHC synapse and auditory fiber damage. The observed severe decline of ribbon numbers, the changes in ABR wave amplitudes and the failure to mobilize Arc in the AC strongly support the idea that increased neuronal activity in the auditory periphery is a compensation of peripheral input – avoiding tinnitus – rather than a correlate or even the origin of tinnitus.
